# Cardiac hydatid cyst: A rare presentation of echinococcal infection 

**DOI:** 10.15171/jcvtr.2019.13

**Published:** 2019-03-13

**Authors:** Ata Firouzi, Mohsen Neshati Pir Borj, Alireza Alizadeh Ghavidel

**Affiliations:** ^1^Cardiovascular Intervention Research Center, Rajaie Cardiovascular Medical and Research Center, Iran University of Medical Sciences, Tehran, Iran; ^2^Rajaie Cardiovascular Medical and Research Center, Iran University of Medical Sciences, Tehran, Iran

**Keywords:** Echinococcal Infection, Hydatid Cyst, Cardiac Echinococcosis

## Abstract

A 57–year-old man presented with atypical chest pain. Transthoracic echocardiography was performed and revealed a very large and well defined intra-myocardial multicystic mass in the posterolateral and basal inferoseptal segments of left ventricle suggestive of hydatid cyst. Although the echocardiographic diagnosis was straightforward, serologic test (hydatid cyst antibody) with enzyme-linked immunosorbent assay (ELISA) was performed which was positive for echinococcal infection. Other works up showed no involvement of other organ system. Albendazol was started for him and he referred to cardiac surgeon for resection of cystic mass.

## Introduction

Echinococcus is an infection caused in human by the larval stage of *Echinococcus granulosus*, *Echinococcus multilocularis*, or *Echinococcus vogeli*. Slowly enlarging echinococcal cyst generally remains asymptomatic until their expanding size or their space occupying effect in an involved organ elicits symptoms. The most pathognomonic finding, if demonstrable is that of daughter cyst within the larger cyst. A specific diagnosis of *E. granulosus* infection can be made by the examination of aspiration fluid for protoscolices, but this is not recommended due to fear of spillage and anaphylactic reactions. Serodiagnostic assays can be useful, although a negative test doses not exclude the diagnosis of echinococcosis. Detection of antibody to specific echinococcal antigen by immunoblotting has the highest degree of specificity. The liver and the lungs are the most common sites of these cysts. Cardiac hydatid cysts are found in fewer than 2% of cases of hydatidosis. In 50% of such cardiac cases, there is multiple organ involvement.^1^

Hydatid cyst of the heart is uncommon and usually develops in the left ventricle. Diagnosis should be considered in patients coming from an endemic area and who present with an abnormal heart shadow on chest x-ray (CXR). The cyst tends to grow and thus compress the neighboring myocardium. It causes displacement of the coronary vessels, rhythm disturbances and mechanical interference with the atrioventricular (AV) valves and ventricular function.^2^

Echocardiography is the imaging method of choice for studying cardiac hydatidosis. Therapy for cardiac echinococcosis is based on consideration of the size, location, and manifestations of cysts and the overall health of the patient. Surgery has traditionally been the principal definitive method of treatment. Albendazol, which is active against echinococcus, should be administered adjunctively, beginning several days before resection and continued for several weeks.

## Case Report

A 57–year-old man, presented with 3 years’ history of atypical chest pain that exaggerated in the past 1 month. He denies any history of weight lost, diaphoresis, fever, rash, gastrointestinal or respiratory symptoms. In His past medical history he had ectatic coronary arteries based on coronary angiography 3 years ago. His Family history and medications or allergies history was negative. In his social history he is married, works as a farmer, he does not use alcohol or illicit drugs and has never smoked. physical examination was Unremarkable. Laboratory data shows positive serologic test (hydatid cyst antibody) with enzyme-linked immunosorbent assay (ELISA), normal liver function tests, renal function tests and complete blood count (CBC) and negative troponin.

Electrocardiography (EKG) shows pathologic Q wave in leads III and aVF and negative T wave in leads II, III, aVF, V6 and left atrium (LA) abnormality.

CXR seems to be normal except prominent aortic knob, LA and left atrial appendage (LAA) enlargement with upturned apex.

**Figure 1:**
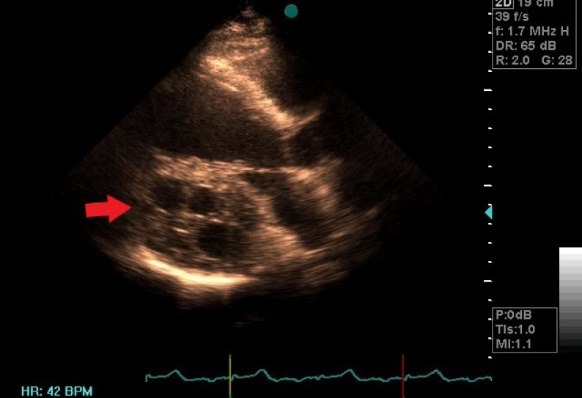
A multicystic mass in left ventricle (Red arrow) seen on TTE in parasternal long axis view.

Transthoracic echocardiography revealed normal left ventricular (LV) size with left ventricular ejection fraction (LVEF): 40%-45%, hypokinesia in mid and base of inferior, inferoseptal and posterior walls secondary to compressive effect of huge mass, mild LV diastolic dysfunction, normal right ventricular (RV) size and systolic function, mild LA enlargement, mild to moderate mitral regurgitation (MR), mild aortic regurgitation (AI), mild pulmonary regurgitation (PI), intact inter atrial septum, no pericardial effusion, a huge, well-defined intramyocardial multicystic mass in the posterolateral and basal inferoseptal segments of left ventricle (Figure 1).

**Figure 2:**
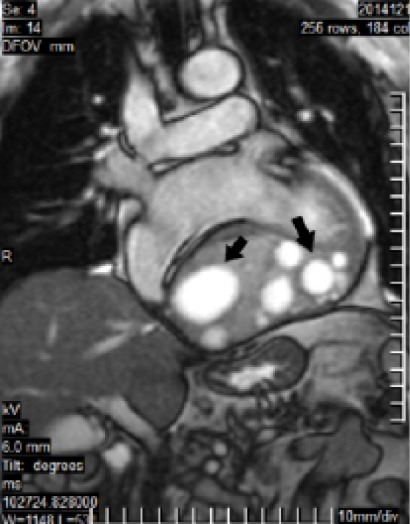
A large cystic left ventricle mass (black arrow) seen on the cardiac MR.

**Figure 3:**
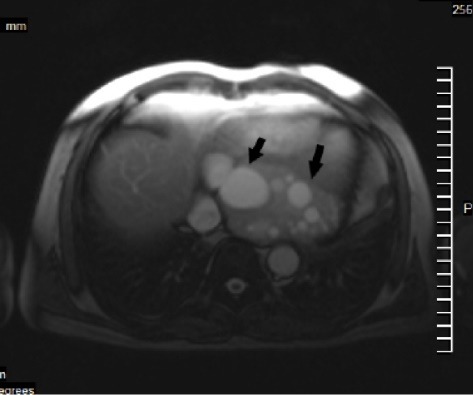
A large cystic left ventricle mass (black arrow) seen on the cardiac MR.

Cardiac MR shows a large encapsulated intramyocardial cystic LV mass which is placed intramyocardium of LV diaphragmatic region and involved basal and mid LV inferior, posterolateral and inferoseptal LV segments (measured 107*75 mm) with evidence of pericystic myocardial fibrosis and include multiple different sizes cysts probably daughter cysts and no obvious evidence of leakage or intracavitary rupture (Figures 2 and 3).

Another cyst placed in the right atrioventricular (AV) groove (27*25 mm) behind right atrium (RA) and predict possibility of mass spreading to pericardial space.

Although the echocardiographic diagnosis was straightforward, transesophageal and 3D echocardiography and cardiac MR was done for better evaluation of mass.

Due to patient complain of exaggerated chest pain from 1 month ago and based on EKG, coronary angiography was performed that revealed ecstatic coronary arteries, single vessel disease (OM 1 was totally cut after branching).

Abdominal CT scan with contrast showed a large soft tissue mass (120 * 70 mm) with heterogeneous pattern and hypodense foci probably cystic form in lower mediastinum and adjacent to posterior aspect of the heart that extended to fundus of stomach inferiorly. Liver and spleen were normal in density. Further studies showed no involvement of other organ systems. Albendazol was administered 1 week before surgery and continued up to 3 months after surgery.

**Figure 4:**
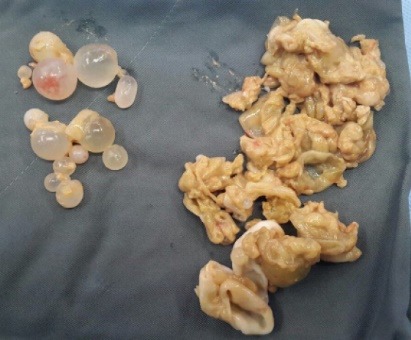
Ruptured cysts and intact daughter cysts.

Cardiopulmonary bypass was used due to the least risk of spillage of cyst contents during the procedure and sponges soaked with hypertonic saline solution were distributed throughout the pericardial cavity to prevent local invasion by the parasite. A lot of ruptured cysts and intact daughter cysts were removed by surgeon and the remaining holes were filled with scolicidal agent (Figure 4). Intraoperative transesophageal echocardiography (TEE) showed complete removal of hydatid cysts. Pathological examination confirmed the diagnosis of hydatid cyst.

## Discussion

Cardiac echinococcosis is scarcely encountered with a frequency of 0.01% to 2%.^3^ Because contractions of the heart provide a natural resistance to the presence of viable hydatid cyst, primary echinococcosis of the heart is a rare event.^4^ Although any part of the heart may be affected, the most common location is the free wall of left ventricle (50%-77%)^5^ or interventricular septal wall^6^ followed by atria and intracavity area.^7^ The disease can remain asymptomatic (90%) but may incidentally result in heart failure, cardiac tamponade, pulmonary embolism, stroke, atrioventricular block, paroxysmal supraventricular tachycardia, mitral regurgitation, pericardial effusion, coronary artery disease, anaphylaxis and death. Diagnosis of cardiac hydatid cyst is easy with typical cystic appearance in echocardiography; however, it may rarely be difficult to distinguish it from myxoma.^8,9^

Coronary narrowing may occur secondary to compression by hydatid cyst^10^ as in our case OM branch was involved probably due to pressure effect of the hydatid cyst.

Transthoracic echocardiography showing the cyst with echo negative contents and smooth contours is the most efficient method of diagnosing the hydatid cyst.^11^ Other diagnostic steps to be taken subsequently include CT scan and MRI and performance of serologic tests. Because of the localization in myocardium, pericardium and life threatening complication, aggressive treatment is deemed necessary. Early excision with standby cardiopulmonary bypass is advisable with albendazol to be administered as an anti-echinococcus medication.

## Ethical approval

All the patient’s data were confidential.

## Competing interests

None.

## Acknowledgments

This work was financially supported by Vice Chancellor for Research, Rajaie Cardiovascular, Medical and Research Center.
